# Characterization of neurotropic HPAI H5N1 viruses with novel genome constellations and mammalian adaptive mutations in free-living mesocarnivores in Canada

**DOI:** 10.1080/22221751.2023.2186608

**Published:** 2023-03-15

**Authors:** Tamiru N. Alkie, Sherri Cox, Carissa Embury-Hyatt, Brian Stevens, Neil Pople, Margo J. Pybus, Wanhong Xu, Tamiko Hisanaga, Matthew Suderman, Janice Koziuk, Peter Kruczkiewicz, Hoang Hai Nguyen, Mathew Fisher, Oliver Lung, Cassidy N. G. Erdelyan, Orie Hochman, Davor Ojkic, Carmencita Yason, Maria Bravo-Araya, Laura Bourque, Trent K. Bollinger, Catherine Soos, Jolene Giacinti, Jennifer Provencher, Sarah Ogilvie, Amanda Clark, Robyn MacPhee, Glen J. Parsons, Hazel Eaglesome, Sayrah Gilbert, Kelsey Saboraki, Richard Davis, Alexandra Jerao, Matthew Ginn, Megan E.B. Jones, Yohannes Berhane

**Affiliations:** a National Centre for Foreign Animal Disease, Canadian Food Inspection Agency, Winnipeg, Canada; b College of Biological Science, University of Guelph, Guelph, Canada; c Canadian Wildlife Health Cooperative, Guelph, Canada; d Veterinary Diagnostic Services, Manitoba Agriculture, Winnipeg, Canada; e Fish and Wildlife, Alberta Environment and Parks, Edmonton, Canada; f Department of Biological Sciences, University of Alberta, Edmonton, Canada; g Animal Health Laboratory, University of Guelph, Guelph, Canada; h Atlantic Veterinary College, University of Prince Edward Island, Charlottetown, Canada; i University of Saskatchewan, Saskatoon, Canada; j Canadian Wildlife Health Cooperative, Atlantic Region, Charlottetown, Canada; k Department of Veterinary Pathology, Western College of Veterinary Medicine, University of Saskatchewan, Saskatoon, Canada; l Environment and Climate Change Canada, Saskatoon, Canada; m Environment and Climate Change Canada, Ottawa, Canada; n Department of Pathology and Microbiology, Atlantic Veterinary College, University of Prince Edward Island, Charlottetown, Canada; o Nova Scotia Department of Natural Resources and Renewables, Kentville, Canada; p National Wildlife Centre, Caledon, Canada; q Wildlife Haven Rehabilitation Centre, Île-des-Chênes, Canada; r Fish and Wildlife Branch, Manitoba Natural Resources and Northern Development, Gimli, Canada; s Office of the Chief Veterinarian, Manitoba Agriculture, Winnipeg, Canada; t Prince Edward Island Department of Environment, Energy and Climate Action, Charlottetown, Canada; u Department of Animal Science, University of Manitoba, Winnipeg, Canada

**Keywords:** Clade 2.3.4.4b, HPAI, H5N1, reassortment, mutation, mammals

## Abstract

The GsGd lineage (A/goose/Guangdong/1/1996) H5N1 virus was introduced to Canada in 2021/2022 through the Atlantic and East Asia-Australasia/Pacific flyways by migratory birds. This was followed by unprecedented outbreaks affecting domestic and wild birds, with spillover into other animals. Here, we report sporadic cases of H5N1 in 40 free-living mesocarnivore species such as red foxes, striped skunks, and mink in Canada. The clinical presentations of the disease in mesocarnivores were consistent with central nervous system infection. This was supported by the presence of microscopic lesions and the presence of abundant IAV antigen by immunohistochemistry. Some red foxes that survived clinical infection developed anti-H5N1 antibodies. Phylogenetically, the H5N1 viruses from the mesocarnivore species belonged to clade 2.3.4.4b and had four different genome constellation patterns. The first group of viruses had wholly Eurasian (EA) genome segments. The other three groups were reassortant viruses containing genome segments derived from both North American (NAm) and EA influenza A viruses. Almost 17 percent of the H5N1 viruses had mammalian adaptive mutations (E627 K, E627V and D701N) in the polymerase basic protein 2 (PB2) subunit of the RNA polymerase complex. Other mutations that may favour adaptation to mammalian hosts were also present in other internal gene segments. The detection of these critical mutations in a large number of mammals within short duration after virus introduction inevitably highlights the need for continually monitoring and assessing mammalian-origin H5N1 clade 2.3.4.4b viruses for adaptive mutations, which potentially can facilitate virus replication, horizontal transmission and posing pandemic risks for humans.

## Introduction

Influenza A viruses (IAVs) are segmented and enveloped RNA viruses [1,2]. Each mature virion of IAVs encases a genome of eight negative-sense single-stranded viral RNA [3], which encode at least 13 proteins [4]. Most avian influenza viruses of the H5 and H7 subtypes with different combinations of neuraminidase (NA) proteins are low pathogenic avian influenza (LPAI) viruses to gallinaceous avian species. However, upon circulation in gallinaceous domestic poultry, some of the H5 and H7 subtypes can evolve to become highly pathogenic after acquiring multiple basic amino acid sequences in the hemagglutinin cleavage site [5,6]. The Eurasian GsGd lineage H5N1 virus that emerged in a rural area in Southern China in 1996 and spread across Asia, Europe, Africa, and North America is typically highly pathogenic for poultry and for some wild birds [7,8]. Over time, the GsGd lineage H5 viruses attained a higher degree of pathogenicity and expanded their host ranges through antigenic drift and multiple rounds of antigenic shift that involved reassortment of various genome segments [9,10].

H5Nx viruses belonging to clade 2.3.4.4 viruses and their different reassortant variants are the major causes of large-scale influenza outbreaks in poultry and wild birds worldwide [11,12]. In Europe, the reassortant H5N8 clade 2.3.4.4 alone caused multiple outbreaks with larger epidemics in 2014/2015, 2016/2017, and 2020/2021 [13]. In North America, the HPAI H5N8 (clade 2.3.4.4c) viruses reassorted with NAm lineage LPAI viruses circulating in wild birds to give rise to two reassortant HPAI viruses of the H5N2 and H5N1 subtypes. The H5N2 virus was mostly responsible for large-scale HPAI outbreaks in the USA and Canada between 2014 and 2015 [14,15]. In the 2021/2022 HPAI H5N1 outbreaks in North America, the number of animal species involved in the outbreaks far exceeded that of the 2014/2015 outbreaks [16,17]. In contrast to the 2014/2015 outbreak, which started on the Pacific coast, the 2021/2022 outbreak started on the Atlantic coast of Canada and inflicted higher levels of mortality in domestic and wild bird species. A significant proportion of mortality in the current outbreaks involved waterfowl, water-associated birds, and different species of raptors.

Isolated cases of influenza infection associated with GsGd lineage H5Nx viruses were also documented in terrestrial mammals such as domestic dogs and cats [18,19], tigers and leopards [20,21], and foxes [22] in a few countries. Despite the sporadic cases of detection of H5Nx viruses in mammals, there was no evidence of sustained transmission between the mammalian species. However in some cases, the presence of specific amino acid residues associated with adaptation to mammalian hosts was observed [17]. In one experimental infection study using ferrets, efficient in-contact transmission between individual animals was observed after exposure to emergent H5N6 viruses [23]. Infections with HPAI H5Nx viruses have been reported more commonly in domestic and captive mammalian species than in free-living species [21,24]. The naturally infected animals, whether domestic, captive, or free-living, showed respiratory illness and systemic involvement of the central nervous system (CNS), but some animals remained subclinical [25,26].

This study describes the presence of wholly Eurasian and three novel reassortant H5N1 clade 2.3.4.4b viruses with various genome constellations in free-living mesocarnivore species (red foxes, striped skunks and American mink) in Canada in 2022. The viruses characterized from the mesocarnivore species had similar genetic compositions with H5N1 viruses circulating in avian species in the same geographic areas. The spillover of these viruses from wild birds to mammals could cause a potentially devastating pandemic if the H5N1 viruses mutate into forms that can spread efficiently among the mammalian species.

## Materials and methods

### Clinical samples from wild mammals

Various samples including oropharyngeal swabs, lung, brain, and other tissues were collected between 12 April 2022 and 29 July 2022 from mesocarnivore species that were found dead or euthanized at various rehabilitation centres or at the wildlife clinics by Canadian Wildlife Health Cooperative regional centres. Clinical samples collected from animals with neurological disease were first tested for rabies and canine distemper at regional Animal Health Laboratories or provincial Canadian Animal Health Network Labs. Samples that tested negative for rabies and canine distemper were further tested for the presence of IAVs. All IAV H5-positive samples were submitted to the National Centre for Foreign Animal Disease (NCFAD) laboratory in Winnipeg for confirmatory testing. Clinical histories of all the cases involving mesocarnivore species used in this study are summarized in Table 1.

**Table 1.** Clinical history of red foxes, skunks and mink naturally infected with various HPAI H5N1 viruses.

GISAID_ID	Virus Name	Provinces	Reassortment patterns				Clinical status
			Pattern 1*	Pattern 2^+^	Pattern 3^#^	Pattern 4^$^	
EPI_ISL_16014529	A/Red Fox/ON/FAV-0558-02/2022	ON		X			A kit with foaming at the mouth, Died on arrival at a rehab center
EPI_ISL_16014527	A/Red Fox/ON/FAV-0558-01/2022	ON		X			Severe ataxia, seizures, unable to carry head up. Died within 2 h of arrival at a wildlife sanctuary
EPI_ISL_16014448	A/Red Fox/ON/FAV-0531-01/2022	ON		X			Progressive seizures, died in a rehabilitation center
EPI_ISL_16014445	A/Red Fox/ON/FAV-0300-06/2022	ON		X			A kit with seizures, unconsciousness
EPI_ISL_16014368	A/Red Fox/ON/FAV-0300-05/2022	ON		X			A kit with seizures, unconsciousness
EPI_ISL_16014112	A/Red Fox/ON/FAV-0300-01/2022	ON		X			Found dead on rail trails
EPI_ISL_16013993	A/Red Fox/ON/FAV-0873/2022	ON		X			Progressive seizures, euthanized
EPI_ISL_16013990	A/Red Fox/ON/FAV-0773-01/2022	ON		X			Found dead
EPI_ISL_16013987	A/Red Fox/ON/FAV-0529-03/2022	ON		X			Found dead
EPI_ISL_16013986	A/Red Fox/ON/FAV-0529-02/2022	ON		X			Neurologic signs, euthanized
EPI_ISL_16003281	A/Red Fox/ON/FAV-0605-03/2022	ON		X			Lethargic, seizures, euthanized
EPI_ISL_16003275	A/Red Fox/ON/FAV-0605-01/2022	ON		X			Neurologic signs, died on arrival at the wildlife refuge
EPI_ISL_16003270	A/Red Fox/ON/FAV-0605-02/2022	ON		X			Found dead
EPI_ISL_16013982	A/Wild Mink/ON/FAV-0529-01/2022	ON		X			Seizures, tremor
EPI_ISL_16021854	A/Red Fox/NS/FAV-0879/2022	NS	X				No history available
EPI_ISL_16021788	A/Red Fox/NS/FAV-0543/2022	NS	X				Seizures
EPI_ISL_16021587	A/Red Fox/PEI/FAV-0591-02/2022	PEI	X				Found dead
EPI_ISL_16020773	A/Red Fox/PEI/FAV-0544/2022	PEI	X				A kit with seizures, tremor, marked lethargy
EPI_ISL_16020774	A/Red Fox/PEI/FAV-0592-01/2022	PEI	X				A kit with seizures, tremor
EPI_ISL_16367971	A/Skunk/SK/FAV-0824-01/2022	SK				X	Found dead
EPI_ISL_16022233	A/Red Fox/SK/FAV-0548-03/2022	SK				X	Exhibiting seizures
EPI_ISL_16367899	A/Red Fox/SK/FAV-0824-96/2022	SK				X	Found dead
EPI_ISL_16023453	A/Skunk/AB/FAV-0416-03/2022	AB			X		Ataxia, seizures, rolling on the ground
EPI_ISL_16023452	A/Skunk/AB/FAV-0416-02/2022	AB			X		Ataxia, severe seizures, rolling
EPI_ISL_16023451	A/Skunk/AB/FAV-0416-01/2022	AB			X		Ataxia, severe seizures
EPI_ISL_16023450	A/Skunk/AB/FAV-0835-10/2022	AB			X		Ataxia, seizures, rolling
EPI_ISL_16023449	A/Skunk/AB/FAV-0835-07/2022	AB			X		Neurologic signs, seizures, rolling, died
EPI_ISL_16023377	A/Skunk/AB/FAV-0835-05/2022	AB			X		Neurologic signs, seizures, rolling, died
EPI_ISL_16023376	A/Skunk/AB/FAV-0835-11/2022	AB				X	Found dead at HPAI virus confirmed poultry premises
EPI_ISL_16023375	A/Skunk/AB/FAV-0897-03/2022	AB				X	Lethargy, progressed to ataxia and seizures
EPI_ISL_16023374	A/Skunk/AB/FAV-0897-02/2022	AB				X	Ataxia, seizures, rolling
EPI_ISL_16023372	A/Skunk/AB/FAV-0897-01/2022	AB				X	Marked lethargy, head tremor, crusty eyes
EPI_ISL_16023371	A/Red Fox/AB/FAV-0835-13/2022	AB				X	Found dead
EPI_ISL_16023370	A/Red Fox/AB/FAV-0835-02/2022	AB				X	Ataxia, tremor, seizures
EPI_ISL_16023345	A/Red Fox/AB/FAV-0835-01/2022	AB				X	Ataxia, severe seizures
EPI_ISL_16003253	A/Red Fox/MB/FAV-0370-01/2022	MB				X	Neurologic signs
EPI_ISL_16003265	A/Red Fox/MB/FAV-0414-04/2022	MB			X		Grand mal seizures, torticollis, ataxia, impaired vision and hearing, dyspnoea, dehydration
EPI_ISL_16003266	A/Red Fox/MB/FAV-0414-08/2022	MB			X		Ataxia, dyspnoea
EPI_ISL_16003267	A/Red Fox/MB/FAV-0414-12/2022	MB			X		Seizures, dyspnoea, dehydration
EPI_ISL_16003268	A/Skunk/MB/FAV-0470/2022	MB			X		Ataxia, head tremor, seizures

*Pattern 1 viruses contain wholly Eurasian H5N1 virus gene segments. **^+^**Pattern 2 reassortant viruses contain gene segments (PB2, PB1, PA, and NP) from NAm lineage IAVs and EA H5N1 viruses (HA, NA, M, and NS). ^#^Pattern 3 viruses are reassortant viruses with PB2 and NP gene segments from NAm IAVs and six other segments (PB1, PA, HA, NA, M, and NS) from EA H5N1 viruses. ^$^Pattern 4 viruses possess PB2, PB1, NP, and NS genes from NAm IAVs and PA, HA, NA, and M genes from EA H5N1 viruses.

ON = Ontario; NS = Nova Scotia; PEI = Prince Edward Island; SK = Saskatchewan; AB = Alberta; MB = Manitoba.

### RNA extraction and virus detection

Total RNA was extracted from clinical specimens (swabs and tissues) and virus isolates using the MagMax 1836 Nucleic Acid Isolation Kit using the KingFisher Duo prime platform (ThermoFisher Scientific, Waltham, MA, USA). The presence of IAV genomic material was verified using the matrix gene-specific real-time RT–PCR followed by H5-specific real-time RT–PCR as described previously [27,28].

### Nanopore sequencing and genome assembly

The full genome segments of IAVs were amplified directly from clinical specimens and isolates using RT–PCR as described [29]. Nanopore sequencing was performed on an Oxford Nanopore GridION sequencer with an R9.4.1 flowcell after library construction using the rapid barcoding kit (SQK-RBK004 or SQK-RBK110.96). The raw Nanopore signal data was basecalled and demultiplexed with Guppy (v5.1.12) using the high accuracy or super-accurate basecalling model on each runs. Basecalled Nanopore reads were analysed and assembled with the CFIA-NCFAD/nf-flu (v3.1.0) Nextflow workflow, which ran: IRMA for initial genome assembly and nucleotide BLAST (v2.12) search of IRMA assembled genome segment sequences against all sequences from the NCBI Influenza Virus Sequence Database (*n* = 959,847) (https://www.ncbi.nlm.nih.gov/genomes/FLU/Database/nph-select.cgi?go) and influenza virus sequences from the 2021/2022 H5N1 outbreaks.

### Phylogenetic analysis

A total of 2364 full genome sequences of IAVs of various subtypes collected between 1 January 2015 and 19 July 2022 from North America and a total of 1318 full genome sequences of highly pathogenic H5N1 clade 2.3.4.4b avian influenza viruses were collected between October 2020 and 19 July 2022 from Africa (*n* = 26), Asia (*n* = 124) and Europe (*n* = 1168) were downloaded from the Global Initiative on Sharing All Influenza Data (GISAID, https://www.gisaid.org). Sequences downloaded from GISAID and generated in our study, 40 from mesocarnivores and 41 from birds, were aligned using MUSCLE, and alignments were trimmed to contain major open reading frames for PB2 (nucleotides [nt] = 2280), PB1 (nt = 2274), PA (nt = 2151), HA (nt = 1704), NP (nt = 1497), and NA (nt = 1410), whereas M and NS segments were trimmed as 982 and 838 nt, respectively. Before selecting representative strains, all sequences of eight segments were separately pooled with sequences generated in this study, and the alignments were subject to maximum likelihood (ML) tree inference using IQ-TREE web server (http://iqtree.cibiv.unvie.ac.at). The best-fitting substitution model for each segment dataset was determined by IQ-TREE and the ultrafast bootstrap with 1000 bootstraps was used for branch support analysis. For the 81 H5N1 full genome sequences generated in this study, a Bayesian phylogenetic analysis was used for concatenated sequences from the coding region of PB2, PB1, PA, HA, NP, NA, M1, M2, NS1, and NS2 using a Bayesian Markov Chain Monte Carlo (BMCMC) method [30] as implemented in the BEAST 2 program (version 2.6.7) [31]. The GTR + G nucleotide substitution model was applied to the data set for Bayesian analysis. The age of the viruses was defined as the date of sample collection from the dataset. The coalescent Bayesian skyline was used for tree prior and an uncorrelated log-normal relaxed clock model was used to reflect the complex population dynamics of H5N1 viruses. For the dataset, at least two independent BEAST analyses runs were conducted for 50 million generations, sampling every 5000 generations. Convergences and effective sample sizes (ESS) of the estimates were checked using Tracer v1.7.2 (http://tree.bio.ed.ac.uk/software/tracer). All parameter estimates for each run showed ESS values > 200. A maximum clade credibility (MCC) phylogenetic tree was generated to summarize all 10,000 trees after a 10% burn-in using TreeAnnotator in BEAST 2 [31]. The time-stamped phylogenetic tree was visualized and annotated using FigTree v1.4.4 (http://tree.bio.ed.ac.uk/software/figtree).

### Antibody detection

Blood samples were collected from red foxes in Ontario that survived clinical disease 3–4 weeks after infection. IAV antibodies in the convalescent sera were detected using an in-house developed IAV nucleoprotein (NP) specific competitive ELISA (cELISA) [32] or H5 HA-specific cELISA [33]. Hemagglutination inhibition (HI) assay was carried out for determining anti-hemagglutinin antibodies. The serum samples were heat inactivated, hemadsorbed, and treated with a receptor-destroying enzyme (RDE) – 4 volumes with 0.1 mL of serum and incubated overnight at 37°C followed by inactivation with 1.5% sodium citrate at 56°C for 30 min. The A/Fancy chicken/NL/FAV-0033/2021 (clade 2.3.4.4b H5N1) was used as an antigen (4HA units) in the HI assay. HI antibody titers were determined as the reciprocal of the highest sample dilution resulting in complete inhibition of the red blood cells from hemagglutination. The resulting HI titers were adjusted to account for the dilution of serum samples with RDE.

### Histological and immunohistochemical evaluation

Brain, lung, and various other tissues collected from red foxes in Ontario, Manitoba, Nova Scotia, and Prince Edward Island (PEI) were processed for hematoxylin and eosin staining for microscopic analysis. Immunohistochemical detection of IAV antigen in tissue sections was conducted as described previously. A mouse monoclonal antibody specific for influenza A nucleoprotein (F26NP9) [32] was applied as a primary antibody. The microscopic sections were then visualized using a horseradish peroxidase-labelled polymer, Envision®^+^ system (anti-mouse) (Dako, USA), and reacted with the chromogen 3,3′-Diaminobenzidine. The sections were counter-stained with Gill’s hematoxylin.

## Results

### Genetic and phylogenetic analysis of H5N1 clade 2.3.4.4b viruses in free-living mesocarnivores

**Figure 1.**
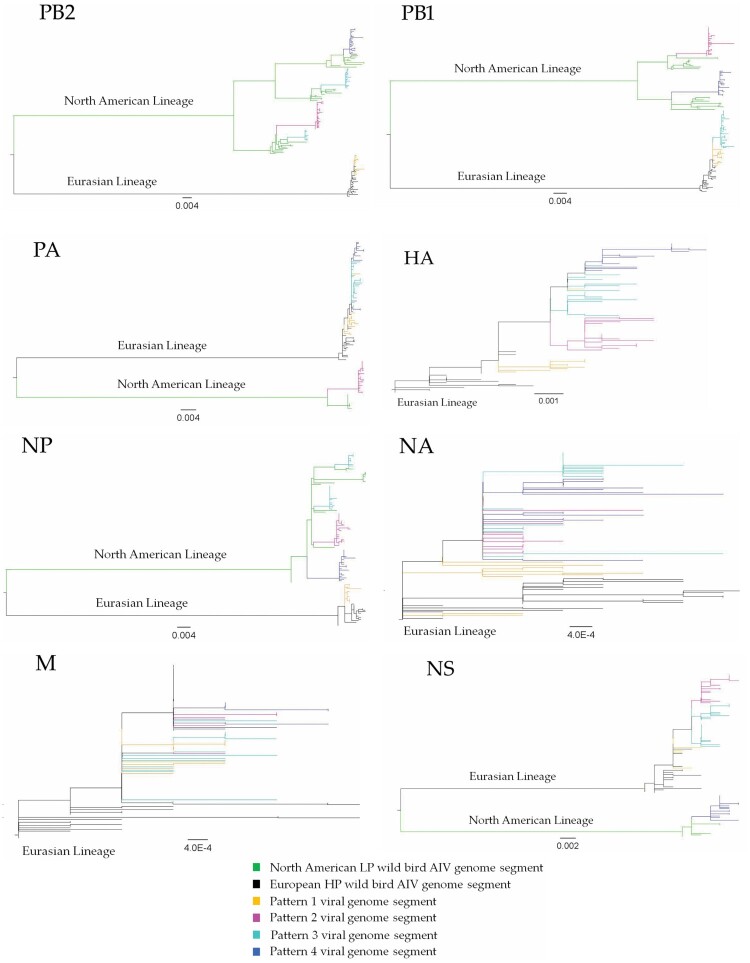
Phylogenetic relationships of highly pathogenic H5N1 viruses of clade 2.3.4.4b. Green branches on the phylogenies represent North American lineage low pathogenic IAVs collected in North America between 2017 and 2021, while black branches denote highly pathogenic H5N1 viruses of clade 2.3.4.4b collected in Europe between October 2020 and November 2021. Highly pathogenic H5N1 viruses of clade 2.3.4.4b collected in Canada between November 2021 and July 2022 are colour coded as follows: yellow = pattern 1 viral genome; magenta = pattern 2 viral genome; cyan = pattern 3 viral genome; and blue = pattern 4 viral genome. Horizontal branch lengths are drawn to scale (nucleotide substitutions per site). Each segment tree is mid-point rooted except for segments HA, NA, and M which are rooted with the oldest sequences in the database.

**Figure 2.**
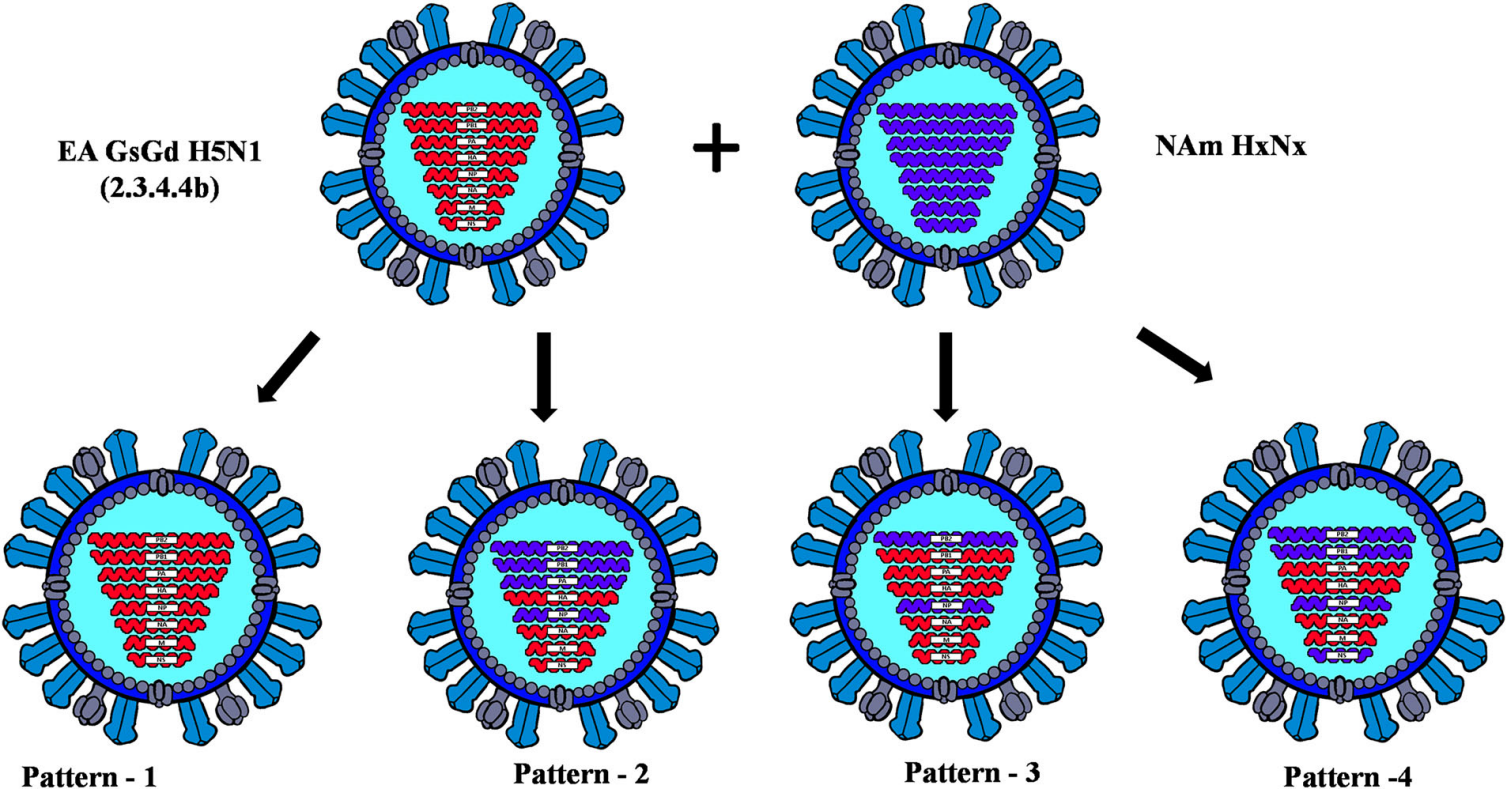
The genome composition and reassortment patterns of HPAI H5N1 (clade 2.3.4.4b) viruses. Virus sequences were obtained from three mesocarnivore species (red foxes, striped skunks, and mink) in Canada in 2022.

To confirm the origin of each EA genome segment of the 40 H5N1 viruses obtained from the mesocarnivore species, phylogenetic tree analysis was conducted for each genome segment individually in comparison with HPAI H5N1 virus sequences deposited at GISAID between October 2020 and 19 July 2022 from Africa, Asia, and Europe. In addition, to confirm the origin of NAm lineage genome segments, IAVs of various subtypes collected from NAm between 1 January 2015 and 19 July 2022 were used. Phylogenetic analyses of the HA gene of viruses originated from Canadian mesocarnivore species (*n* = 40) and wild birds (*n* = 41) showed closer relationships to HPAI H5N1 viruses from Ireland (isolated on 19 November 2021) and Iceland (isolated on 25 October 2021). The same genetic relationships were observed for NA and M segments in all 81 Canadian H5N1 viruses (Figure 1). However, 35 out of 40 H5N1 viruses from mesocarnivore species contained a combination of two or more genome segments (either PB2, PB1, PA, NP, or NS) from NAm lineage LPAI viruses. Only 5 out of 40 viruses possessed wholly EA genome segments. The reassortment patterns of the viruses are presented in Table 1 and illustrated in Figure 2. The full genome sequences of H5N1 viruses (*n* = 81) generated in this study were deposited at the GISAID. ORG database and accession numbers were indicated in Table 1 and Supplementary Table 1.

**Figure 3.**
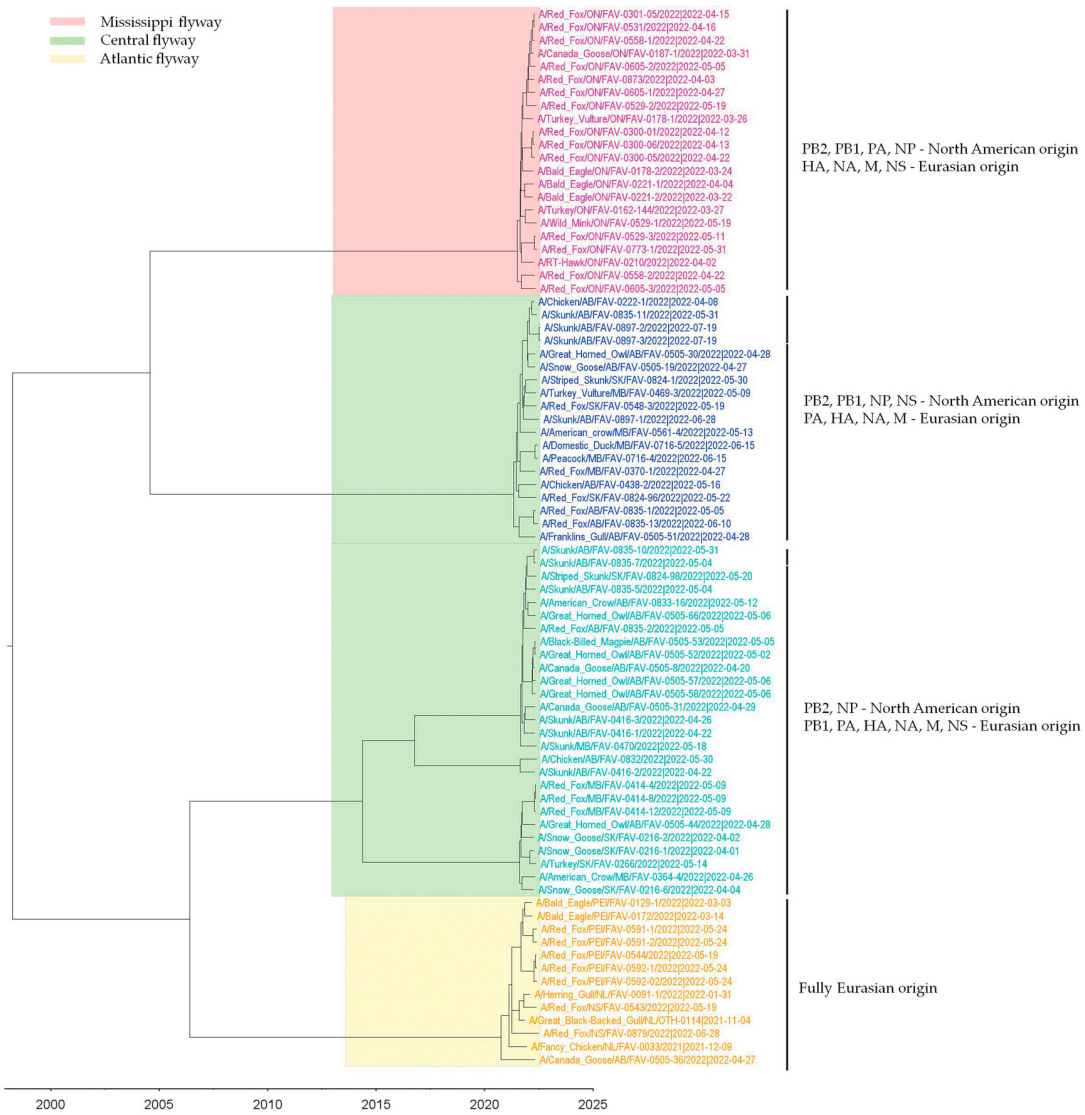
MCC tree inferred using Bayesian MCMC analysis of concatenated full genome sequences of H5N1 clade 2.3.4.4b viruses obtained from mesocarnivores and birds. The clustering evidence of the viruses on the phylogenetic tree demonstrates the genetic relatedness of viruses from red fox, skunks, and mink with avian-origin H5N1 within a specific North American wild bird migration flyways. Branches are highlighted to reflect North American bird migration flyways: yellow for Atlantic flyway; pink for Mississippi flyway; and light green for Central flyway. AB = Alberta; MB = Manitoba; SK = Saskatchewan; ON = Ontario; PEI = Prince Edward Island; NS = Nova Scotia.

**Figure 4.**
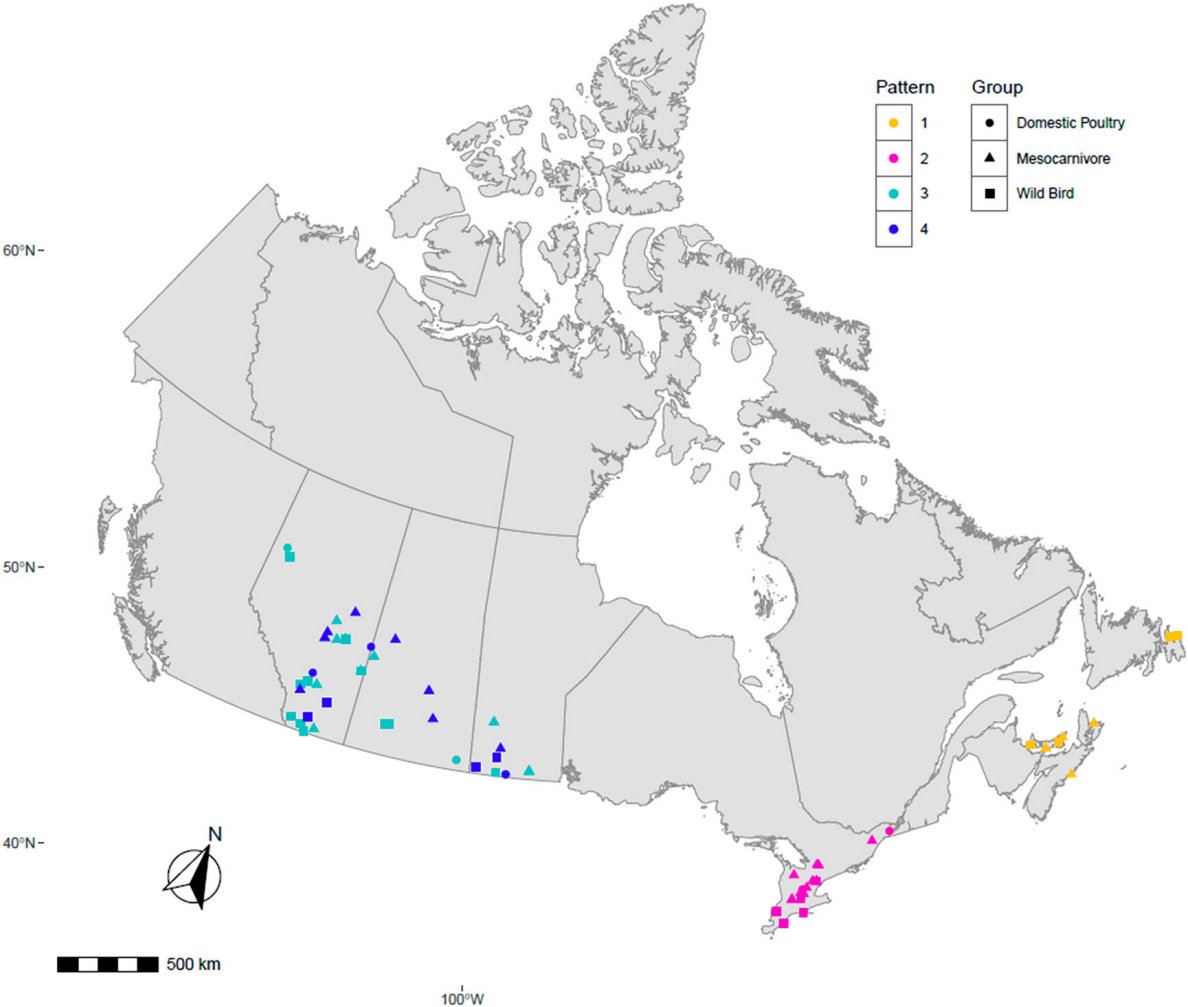
Map describing the geographic location of HPAI H5N1 clade 2.3.4.4b virus obtained from individual mesocarnivore, wild and domestic birds in Canada in 2021/2022. It illustrates viruses with similar reassortment patterns circulating in both mammals, and wild and domestic birds in the exact geographic location. Each shape was plotted on the map based on the exact longitude and latitude coordinates where the animals were found and were plotted on a cartographic boundary shapefile containing the existing provinces and territories of Canada using R programming. H5N1 viruses with the same reassortment pattern circulating in different animals are described using the same colour.

To determine the genetic relationship of H5N1 viruses circulating in birds and mammals in Canada, we generated concatenated sequences from the coding region of PB2, PB1, PA, HA, NP, NA, M1, M2, NS1, and NS2 of 81 H5N1 viruses using a Bayesian Markov Chain Monte Carlo method. Based on this analysis, the H5N1 viruses from the mesocarnivores and birds formed three new genetic clusters based on their reassortment patterns. Interestingly, viruses from both mammals and birds with similar reassortment patterns clustered together and were restricted to specific North American wild bird migratory flyways (Figure 3) in rather localized geographic locations in Canada (Figure 4).

### Analysis of mammalian adaptive mutations

The full genome sequences of H5N1 viruses obtained from foxes, skunks, and mink were assessed for previously known adaptive mutations that may enhance the replication and transmission to other mammalian hosts. All viruses isolated from the mesocarnivores retained some amino acid residues in their hemagglutinin (HA) protein that might enable them to bind to the α-2,6-linked sialic acids. These residues include the substitutions of S137A and T160A that were demonstrated to moderately enhance the binding activity of IAVs to α-2,6-linked sialic acids under *in vitro* studies. Both amino acid substitutions were present in all the H5N1 viruses from mesocarnivores and birds independent of their reassortment patterns and geographic locations. None of the mammalian viruses possessed substitutions in the HA protein such as N186 K, Q226L and G228S (H3 numbering) demonstrated to provide H5 subtype IAVs with strong binding capability to α-2,6 human-like receptors. Significant amino acid substitutions in all the putative antigenic sites of the HA protein were present in all viruses. These substitutions were present in the antigenic site B (A185E, D195 T, V198I), antigenic site C (E268G), and antigen site D (V210A) (H5 numbering) when compared to the A/Gyrfalcon/Washington/41088-6/2014 H5N8 (clade 2.3.4.4c) virus that was previously reported in North America for the first time in 2014/2015.

The majority of H5N1 viruses obtained from mesocarnivores had glutamic acid in their PB2 protein at amino acid position 627 (627E). A couple of red foxes from Atlantic Canada (FAV-0544 and FAV-0592-01) and Ontario (FAV-0558-01) had a substitution of the avian signature glutamic acid (E) to a human signature lysine (K) at position 627 (E627 K). In one of the red foxes (FAV-0592-01) from Prince Edward Island (PEI), a virus originating from the brain sample contained a mixture of the mammalian 627 K and avian 627E signatures, however, the proportion of the mammalian signature was about 66.7%. In contrast, a virus from the lung of the same fox had more of avian signature PB2-627E (87.9%) than the mammalian signature PB2-627 K (10.8%) indicating the occurrence of an adaptive mutation in this specific animal. The PB2 sequence information of a virus originating from the oropharyngeal swabs of this kit’s littermate identified as FAV-0544 had 100% 627 K. Three red foxes from Ontario (FAV-0301-05, FAV-0529-02 and FAV-0531-01) had valine in PB2-627 (E627 V). Another red fox (FAV-0824-096) from Saskachewan had a substitution of D701N in PB2. The other important mutations in the PB2, PB1, PA, and NP segments that have been described to enhance either the polymerase activity or virus replication in mammalian cells are summarized in Table 2. We have identified other unique mutations in PB2, PB1, PA, and NP in viruses isolated from the mesocarnivores species in Canada, however, the biological significances and functions of these amino acid residues are not yet explored.

**Table 2.** Amino acid substitution in the H5N1 viruses associated with adaptation to mesocarnivore species.

Segments	Mutations	Virus property	Viruses obtained from mesocarnivores	References
PB2	T271A	↑polymerase activity in avian and mammalian cells	FAV-0543	[34]
	K389R	↑polymerase activity in mammalian cells	All	[35,36]
	E627K	Enhanced polymerase activity at low temperature, compensates for the lack of 701N, ↑virulence in various mammalian hosts, ↓polymerase activity and replication in avian cells, ↓virulence in chickens	FAV-0544, FAV-0592-01, FAV-0558-01	[Reviewed in 37]
	E627V	Replicate significantly in MDCK cells and human cells	FAV-0529-02, FAV-0531-01, FAV-0301-05	[38]
	L89 V, G309D, T339 K, R477G, I495 V, A676T	Compensates for the lack of 627K	All	[39]
PB1	D3V	↑polymerase activity and replication in mammalian and avian cells	All	[40]
	D622G	↑polymerase activity in mammalian cells ↑virulence in mice	All	[41]
PA	S37A	↑polymerase activity in mammalian cells	All	[35]
	N383D		All	
	N409S	↑polymerase activity and replication in mammalian cells	All	[35]
NP	N319K	↑ polymerase activity and replication in mammalian cells	All	[42,43]

PB2 = polymerase basic protein 2; PB1 = polymerase basic protein 1; PA = polymerase acidic protein; NP = nucleoprotein. ↓decrease, ↑increase.

### Serological findings

Three serum samples collected from red foxes in Ontario that survived H5N1 infection tested positive for the presence of anti-IAV NP antibodies as well as H5 HA-specific antibodies in cELISA tests. On the HI assay using A/Fancy Chicken/NL/FAV-0033/2021 (H5N1 – 2.3.4.4b) antigen, the serum samples obtained from two foxes (FAV-0300-1 and FAV-0300-6) had HI titers of 320, whereas a serum sample from another red fox (FAV-0300-5) had relatively higher HI titer of 640 units.

### Histological and immunohistochemical findings

**Figure 5.**
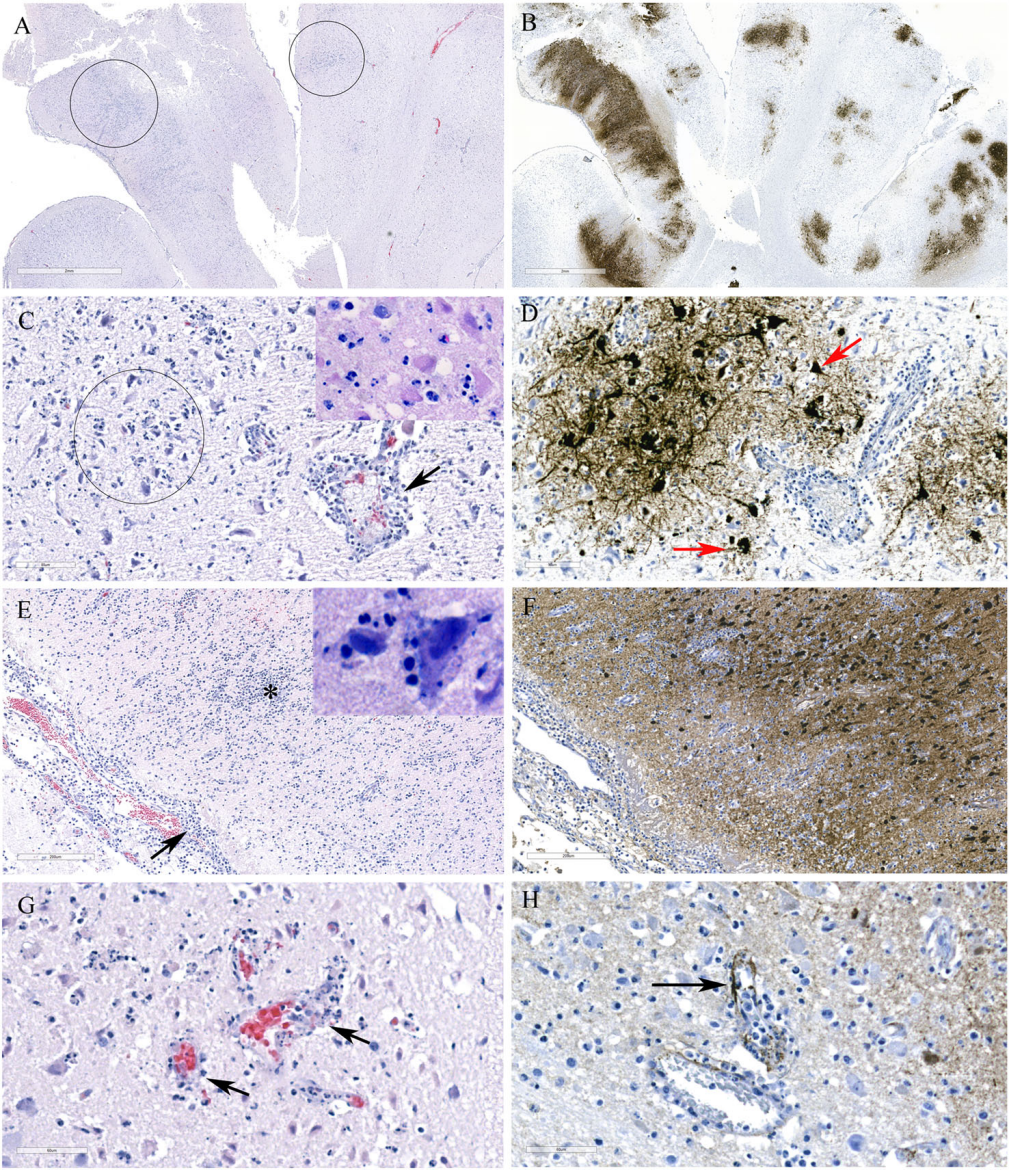
Histopathology and immunohistochemical findings in the brain tissue from a red fox. A. Multifocal areas of necrotizing encephalitis (low magnification, circled). B. Abundant influenza virus antigen (brown deposition) corresponding to areas of inflammation observed in slide A. C. Degeneration of neurons with neutrophil infiltration (circled) and perivascular cuffing with inflammatory cells (black arrow). Inset: Multiple pale eosinophilic neurons with karyolytic nuclei indicating degeneration and presence of neutrophils. D. Abundant viral antigen was detected and corresponds with histologic lesions observed in slide C (red arrows). E. Meningitis (arrow) and areas of increased cellularity due to gliosis and inflammatory cells infiltrate (*). Inset: Neuronophagia. F. Abundant viral antigen in the brain corresponding to areas of inflammation shown in E. G. Vasculitis (arrows). H. Viral antigen detected in endothelial cells (arrow).

**Figure 6.**
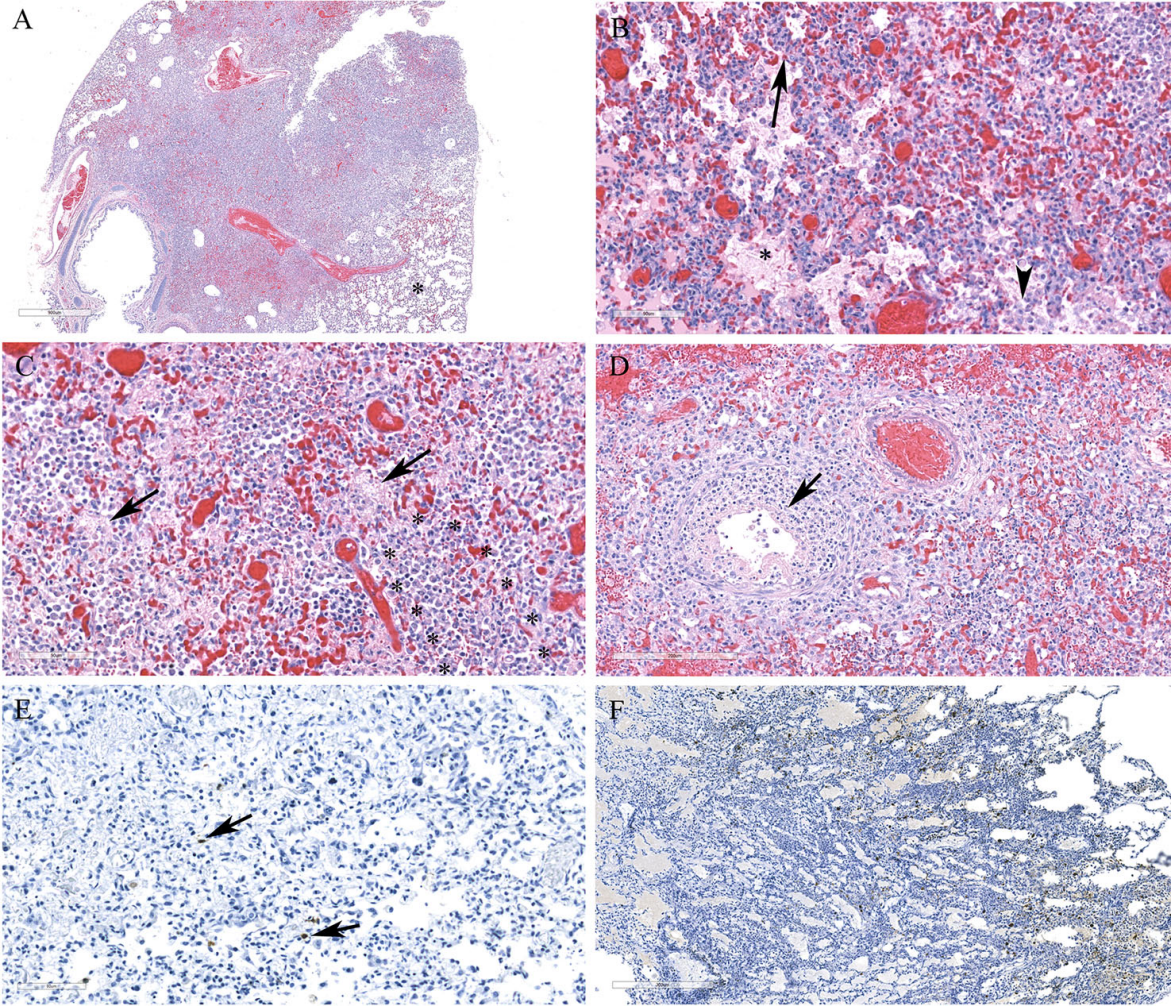
Histopathology and immunohistochemical findings in lung sections from a red fox. A. Extensive pneumonia leading to loss of alveolar spaces. Relatively normal tissue indicated by asterisk (*). B. Interstitial infiltration of inflammatory cells (arrow), alveolar edema (*), increased alveolar macrophages (arrowhead). C. Alveolar spaces contain fibrin (arrows) and neutrophils (outlined by asterisks). D. Necrotizing bronchiolitis (arrow). E. Only occasional cells contain viral antigens from Manitoba samples (arrows). F. Abundant viral antigen detected in a red fox lung sections from Ontario (brown deposits).

Histological lesions in the cerebral cortex of red foxes naturally infected with a reassortant H5N1 virus (FAV-0370-01, a pattern 4 virus from Manitoba) include multifocal necrotic neurons and glial cells, neutrophilic infiltrates into the cortex and perivascular cuffing (Figures 5A and C). Gliosis, neuropil rarefaction, hemorrhage, neuronophagia and meningitis (Figure 5E), and vasculitis (Figure 5G) were present in the brain section. Abundant viral antigen was detected multifocally in all brain structures (Figures 5B and F), however, prominent staining was observed in the neurons and neuropil (Figure 5D). Occasional staining for virus antigens was observed in endothelial cells (Figure 5H). Pneumonic lung lesions (Figure 6A) were characterized by edema, increased alveolar macrophages, and interstitial inflammation (Figure 6B), accumulation of fibrin and necrotic debris in the alveolar spaces and by neutrophilic infiltrates (Figure 6C). Necrotic bronchiolitis (Figure 6D) and a few fibrin microthrombi were detected in the lung section of one of the foxes. The presence of viral antigen was scant, but was detected on the serosa and within the alveolar septa (Figure 6E). The spleen lesions include, severely reduced white pulp and evidence of lymphocytic apoptosis and necrosis, but with no detectable viral antigen. The scant amount of viral antigens was detected in the kidneys and liver despite the absence of visible microscopic lesions (data not shown). Histologic lesions in the brain and lung from red foxes naturally infected with reassortant pattern 2 H5N1 viruses in Ontario and with pattern I viruses in PEI and Nova Scotia resembled the lesions described by a pattern 4 virus, although pneumonia was mild or absent in red foxes affected with pattern I viruses. Abundant viral antigen was detected in the lungs of a red fox from Ontario (Figure 6F). Immunohistochemical evaluation was not performed on tissues from foxes from Nova Scotia and PEI.

## Discussion

Influenza A viruses continuously evolve and as a result accumulate adaptive mutations, which facilitate virus replication, interspecies transmission, and pose pandemic risks for human and animals. In the current study, we examined 40 HPAI H5N1 viruses from clade 2.3.4.4b virus with various genome constellations in multiple mammalian species, notably in mesocarnivore species in Canada. Infections with HPAI H5N1 viruses and mortality in these free-living red foxes, striped skunks, and mink reaffirm that these viruses can overcome interspecies barriers and infect new hosts. The clinical presentation of the disease in red foxes, skunks, and mink was mostly neurologic, resembling clinical and pathologic features described in seals and foxes that were naturally infected with HPAI H5N8 viruses [25]. Oropharyngeal swabs and tissues consistently yielded positive PCR results for IAVs and fox kits that were recovered from the clinical infection developed anti-NP as well as anti HA-specific antibodies. Histologic lesions associated with HPAI H5N1 viruses in a number of red foxes include extensive meningoencephalitis and pneumonia and the presence of a higher load of viral antigen was demonstrated by IHC staining mainly in the brain sections. Reperant et al. (2008) experimentally infected red foxes with wild bird-origin HPAI clade 2.2 H5N1 virus via the intra-tracheal route and through ingestion [26]. Foxes inoculated by the intra-tracheal route excreted viruses orally for a relatively prolonged period and developed severe pneumonia, myocarditis, and encephalitis. Extensive amount of virus antigen was detected in the lung and brain of individuals infected intratracheally with the virus. The foxes that were infected by ingesting wild bird carcasses obtained from experimentally infected wild birds excreted the virus transiently and developed milder pneumonia with traces or undetected virus antigen in the lungs [26]. The observed differences in virus shedding, organ lesions, and virus antigen load between the two modes of experimental infections may likely be related to the amounts of viruses in the inoculum and tissues of birds fed to the foxes. Most infected mammals described in our study are opportunistic mesocarnivore species that were likely exposed to the H5N1 virus by consuming larger quantities of incapacitated or dead birds infected with H5N1 viruses. Fox kits from the same litter, likely shared the same infectious meal. However, horizontal transmission to den mates via aerosol or vertical transmission to kits due to the consumption of milk from a lactating vixen cannot be ruled out. A recent study in the UK provided a clue of possible transmission of HPAI H5N8 virus from swans to red foxes and seals by aerosol means while the birds and the mammalian species were kept in captivity in the same quarantine services [25].

To date, few cases of HPAI H5N1 and H5N8 clade 2.3.4.4b viruses were found in free-living mesocarnivores [22] compared to the relatively common occurrence of HPAI viruses in domestic and captive mammals due to increased accessibility to infected domestic or peridomestic birds [18,22,24]. To our knowledge, this is the first comprehensive report of infection of free-living mesocarnivores with HPAI H5N1 clade 2.3.4.4b viruses in the Americas. Infection in these mammals involved wholly Eurasian 2.3.4.4b H5N1 viruses, and various reassortant viruses with different genome constellations. Similarly, wholly EA and reassortant H5N1 viruses that were detected in the mammalian species were also identified in wild and domestic birds. These are not unexpected spatiotemporal events given the ever-increasing frequency of occurrence and reassortment of H5N1 clade 2.3.4.4b viruses with local LPAI viruses and their persistence in the wild bird population across specific North American migratory bird flyways [44]. The mesocarnivore species are highly mobile by nature, and it is difficult to draw a transmission network of the virus, however, the concatenated phylogenetic tree analysis showed closer relationship of viruses isolated from wild birds and mesocarnivores and viruses of similar genetic cluster was circulating in both local birds and mammals in the same geographic locations demonstrating direct link of infection between both species. At the time of the submission of this manuscript, GsGd lineage HPAI H5 virus sequences were unavailable from free-ranging minks and skunks in North America in the 2021/2022 and previous H5Nx-related outbreaks [45]. However, HPAI H5N1 clade 2.3.2.1b and 2.3.2.1e viruses were isolated from farmed minks in China in 2017 [46].

The full genome sequence analysis of H5N1 viruses derived from red foxes, skunks and mink revealed critical mutations that can affect receptor binding and adaptation to a mammalian host. The mammalian viruses demonstrate significant antigenic drift in their HA protein compared to A/Gyrfalcon/Washington/41088-6/2014 (H5N8) – a zoonotic candidate vaccine virus selected after the 2014/2015 outbreak in North America. The mammalian and avian viruses in the 2021/2022 HPAI virus outbreaks in Canada have substitutions at S137A and T160A in their hemagglutinin that may provide a degree of binding preference to the α-2,6-linked sialic acids as indicated under *in vitro* studies [47]. However, all H5N1 viruses in the current outbreaks lack Q226L and G228S (H3 numbering) substitutions in their HA protein that are expected to confer strong binding efficiency to the α-2,6 human-like receptors that facilitate infections through direct contact in mammals such as ferrets [23].

The replication of H5N1 viruses in mammalian cells is influenced by the polymerase complex activity; among which PB2 plays a main role in virulence [48]. The majority of H5N1 viruses sequenced from the mesocarnivore species retained an avian signature glutamic acid at position 627 of their PB2 (627E) protein. However, HPAI H5N1 from two red foxes from Atlantic Canada and one from Ontario had E627 K substitution in their PB2, whereas three red foxes from Ontario had E627 V substitution. Of the 120 PB2 sequences (available since 2003 in the GISAID database and accessed on 11 July 2022) originated from GsGd lineage HPAI H5Nx viruses from mammalian hosts (excluding humans), 21 sequences only had E627 K and one had E627 V substitutions. From the NCBI database, we were able to extract quite few GsGd H5 viruses (*n* = 15) with the E627 K mutation in their PB2 gene. E627 K mutation was also observed by deep sequencing of H7N9 viruses from oropharyngeal swabs originated from a number of human patients [49] and in the brain samples from harbour seals [50]. E627 V mutation, although is rare, H7N9 viruses from oropharyngeal swabs from human patients carry this particular mutation [49]. E627 K in H5N1 and H7N9 subtypes increased the replication of the viruses *in vitro* in mammalian cells and in mammalian hosts [49] and have led to increased virulence in mammalian hosts [48]. A clade 2.3.4.4b H5N6 virus isolated from a human patient carrying E627 K produced severe pneumonia in intratracheally inoculated ferrets [51]. The mechanism of enhanced pathogenicity of viruses carrying PB2-627 K substitution is believed due to increased polymerase catalytic activity of these viruses at low temperature in the human or mammalian upper respiratory tracts [52]. As well this particular substitution can increase charged surface residues providing increased molecular association of the PB2 C-terminus region with the cytoplasmic importins such as with importin α1 for PB2 to be independently translocated into the nucleus [53,54] of infected cells and therefore, enhance virus replication in mammalian cells [55]. Viruses with PB2-627 K can also impair the nucleocapsid destabilizing effects of innate immune genes including RIG-I and as such viruses carrying this mutation substantially evade innate antiviral immunity in mammals [56]. A mutation of E627 V in PB2 allowed recombinant H5N1 virus to adapt and replicate significantly in MDCK cells and human cells [38]. The polymerase activity of the H9N2 subtype carrying this residue was higher in human cells and enhanced virulence in mice [57]. Influenza viruses with this unique rare mutation may be transmitted and stably maintained in both avian and mammalian species due to the intermediate nature of Valine compared to Lysine and Glutamic acid residues [58]. A HPAI H5Nx sequence originating from a tiger had mutation of E627 V in the PB2. Another less-described substitution in PB2 at position T/I271A with the potential to increase the pathogenicity of clade 2.3.4.4b H5Nx viruses in mice [34] are preserved in at least one of the viruses from a red fox. One red fox in this study had D701N substitution in PB2, such substitution was previously demonstrated to alter virulence [59,60] and was implicated in expanding the host ranges of avian influenza viruses [59]. PB2 sequence data from a fox and a seal in England revealed a substitution of D701N [25]. The viruses carrying a combination of virulence determinants may still have adaptive fitness to replicate in a range of hosts. It remains to be seen whether these unique mutations are correlated with disease severity and reflect a zoonotic potential. Further risk assessment studies are needed to determine the risk these viruses might pose to human health.

## Supplementary Material

Supplemental Material
