# Supplementary material for: Characterization of neurotropic HPAI H5N1 viruses with novel genome constellations and mammalian adaptive mutations in free-living mesocarnivores in Canada
Source: Emerg Microbes Infect. 2023 Mar 15;12(1):2186608. doi: 10.1080/22221751.2023.2186608 (PMC10026807; doi:10.1080/22221751.2023.2186608)
Supplement: Supplemental Material [file TEMI_A_2186608_SM1153.docx]

Supplementary Table 1. HPAI H5N1 viruses isolated from wild birds and poultry in Canada in 2021/2022. GISAID accession numbers are provided.

| **GISAID_ID** | **Isolate_Name** | **Hosts** |
| --- | --- | --- |
| [EPI_ISL_16173525](https://platform.epicov.org/epi3/start/EPI_ISL/16173525) | A/Snow Goose/SK/FAV-0216-06/2022 | *Anser caerulescens* |
| [EPI_ISL_16172175](https://platform.epicov.org/epi3/start/EPI_ISL/16172175) | A/Snow Goose/SK/FAV-0216-02/2022 | *Anser caerulescens* |
| [EPI_ISL_16171530](https://platform.epicov.org/epi3/start/EPI_ISL/16171530) | A/Snow Goose/SK/FAV-0216-01/2022 | *Anser caerulescens* |
| [EPI_ISL_16190850](https://platform.epicov.org/epi3/start/EPI_ISL/16190850) | A/Herring Gull/NL/FAV-0091-01/2022 | *Larus argentatus* |
| [EPI_ISL_16190849](https://platform.epicov.org/epi3/start/EPI_ISL/16190849) | A/Great Black-Backed Gull/NL/OTH-0114/2021 | *Larus marinus* |
| [EPI_ISL_16189981](https://platform.epicov.org/epi3/start/EPI_ISL/16189981) | A/Franklin's Gull/AB/FAV-0505-51/2022 | Leucophaeus |
| [EPI_ISL_16171528](https://platform.epicov.org/epi3/start/EPI_ISL/16171528) | A/Turkey/SK/FAV-0266/2022 | Turkey |
| [EPI_ISL_16189977](https://platform.epicov.org/epi3/start/EPI_ISL/16189977) | A/Great Horned Owl/AB/FAV-0505-30/2022 | Other avian |
| [EPI_ISL_16190394](https://platform.epicov.org/epi3/start/EPI_ISL/16190394) | A/Black-Billed Magpie/AB/FAV-0505-53/2022 | Black billed Magpie |
| [EPI_ISL_16173537](https://platform.epicov.org/epi3/start/EPI_ISL/16173537) | A/Canada Goose/ON/FAV-0187-01/2022 | *Branta canadensis* |
| [EPI_ISL_16189979](https://platform.epicov.org/epi3/start/EPI_ISL/16189979) | A/Canada Goose/AB/FAV-0505-36/2022 | *Branta canadensis* |
| [EPI_ISL_16189978](https://platform.epicov.org/epi3/start/EPI_ISL/16189978) | A/Canada Goose/AB/FAV-0505-31/2022 | *Branta canadensis* |
| [EPI_ISL_16183625](https://platform.epicov.org/epi3/start/EPI_ISL/16183625) | A/Canada Goose/AB/FAV-0505-08/2022 | *Branta canadensis* |
| [EPI_ISL_16174374](https://platform.epicov.org/epi3/start/EPI_ISL/16174374) | A/RT-Hawk/ON/FAV-0210/2022 | Wild bird |
| [EPI_ISL_16173531](https://platform.epicov.org/epi3/start/EPI_ISL/16173531) | A/Turkey Vulture/ON/FAV-0178-01/2022 | Wild bird |
| [EPI_ISL_16190761](https://platform.epicov.org/epi3/start/EPI_ISL/16190761) | A/American Crow/AB/FAV-0833-16/2022 | Wild bird |
| [EPI_ISL_16190578](https://platform.epicov.org/epi3/start/EPI_ISL/16190578) | A/Great Horned Owl/AB/FAV-0505-58/2022 | Wild bird |
| [EPI_ISL_16190395](https://platform.epicov.org/epi3/start/EPI_ISL/16190395) | A/Great Horned Owl/AB/FAV-0505-57/2022 | Wild bird |
| [EPI_ISL_16189983](https://platform.epicov.org/epi3/start/EPI_ISL/16189983) | A/Great Horned Owl/AB/FAV-0505-52/2022 | Wild bird |
| [EPI_ISL_16189980](https://platform.epicov.org/epi3/start/EPI_ISL/16189980) | A/Great Horned Owl/AB/FAV-0505-44/2022 | Wild bird |
| [EPI_ISL_16189912](https://platform.epicov.org/epi3/start/EPI_ISL/16189912) | A/Great Horned Owl/AB/FAV-0505-19/2022 | Wild bird |
| [EPI_ISL_16173532](https://platform.epicov.org/epi3/start/EPI_ISL/16173532) | A/Bald Eagle/ON/FAV-0178-02/2022 | *Haliaeetus leucocephalus* |
| [EPI_ISL_16173529](https://platform.epicov.org/epi3/start/EPI_ISL/16173529) | A/Bald Eagle/ON/FAV-0221-02/2022 | *Haliaeetus leucocephalus* |
| [EPI_ISL_16173528](https://platform.epicov.org/epi3/start/EPI_ISL/16173528) | A/Bald Eagle/ON/FAV-0221-01/2022 | *Haliaeetus leucocephalus* |
| [EPI_ISL_16173527](https://platform.epicov.org/epi3/start/EPI_ISL/16173527) | A/Bald Eagle/PEI/FAV-0172/2022 | *Haliaeetus leucocephalus* |
| [EPI_ISL_16173526](https://platform.epicov.org/epi3/start/EPI_ISL/16173526) | A/Bald Eagle/PEI/FAV-0129-01/2022 | *Haliaeetus leucocephalus* |
| [EPI_ISL_16173549](https://platform.epicov.org/epi3/start/EPI_ISL/16173549) | A/Chicken/ON/FAV-0217-05/2022 | *Gallus gallus domesticus* |
| [EPI_ISL_16173545](https://platform.epicov.org/epi3/start/EPI_ISL/16173545) | A/Chicken/ON/FAV-0208-144/2022 | *Gallus gallus domesticus* |
| [EPI_ISL_16173542](https://platform.epicov.org/epi3/start/EPI_ISL/16173542) | A/Chicken/ON/FAV-0208-133/2022 | *Gallus gallus domesticus* |
| [EPI_ISL_16190673](https://platform.epicov.org/epi3/start/EPI_ISL/16190673) | A/Chicken/AB/FAV-0832/2022 | *Gallus gallus domesticus* |
| [EPI_ISL_16183595](https://platform.epicov.org/epi3/start/EPI_ISL/16183595) | A/Chicken/AB/FAV-0438-02/2022 | *Gallus gallus domesticus* |
| [EPI_ISL_16183504](https://platform.epicov.org/epi3/start/EPI_ISL/16183504) | A/Chicken/AB/FAV-0222-01/2022 | *Gallus gallus domesticus* |
| [EPI_ISL_16173530](https://platform.epicov.org/epi3/start/EPI_ISL/16173530) | A/Turkey/ON/FAV-0162-144/2022 | *Meleagris gallopavo* |
